# Modulation of P2Y_6_R expression exacerbates pressure overload-induced cardiac remodeling in mice

**DOI:** 10.1038/s41598-020-70956-5

**Published:** 2020-08-18

**Authors:** Kakeru Shimoda, Akiyuki Nishimura, Caroline Sunggip, Tomoya Ito, Kazuhiro Nishiyama, Yuri Kato, Tomohiro Tanaka, Hidetoshi Tozaki-Saitoh, Makoto Tsuda, Motohiro Nishida

**Affiliations:** 1 National Institute for Physiological Sciences (NIPS), National Institutes of Natural Sciences, Okazaki, Aichi 444-8787 Japan; 2 Exploratory Research Center on Life and Living Systems (ExCELLS), National Institutes of Natural Sciences, Okazaki, Aichi 444-8787 Japan; 3 SOKENDAI (School of Life Science, The Graduate University for Advanced Studies), Okazaki, Aichi 444-8787 Japan; 4 Graduate School of Pharmaceutical Sciences, Kyushu University, 3-1-1 Maidashi, Higashi-ku, Fukuoka, 812-8582 Japan; 5 Faculty of Medicine and Health Sciences, University Malaysia Sabah, 88400 Kota Kinabalu, Sabah Malaysia; 6 Center for Novel Science Initiatives (CNSI), National Institutes of Natural Sciences, Tokyo, 105-0001 Japan

**Keywords:** Heart failure, Cardiac hypertrophy

## Abstract

Cardiac tissue remodeling caused by hemodynamic overload is a major clinical outcome of heart failure. Uridine-responsive purinergic P2Y_6_ receptor (P2Y_6_R) contributes to the progression of cardiovascular remodeling in rodents, but it is not known whether inhibition of P2Y_6_R prevents or promotes heart failure. We demonstrate that inhibition of P2Y_6_R promotes pressure overload-induced sudden death and heart failure in mice. In neonatal cardiomyocytes, knockdown of P2Y_6_R significantly attenuated hypertrophic growth and cell death caused by hypotonic stimulation, indicating the involvement of P2Y_6_R in mechanical stress-induced myocardial dysfunction. Unexpectedly, compared with wild-type mice, deletion of P2Y_6_R promoted pressure overload-induced sudden death, as well as cardiac remodeling and dysfunction. Mice with cardiomyocyte-specific overexpression of P2Y_6_R also exhibited cardiac dysfunction and severe fibrosis. In contrast, P2Y_6_R deletion had little impact on oxidative stress-mediated cardiac dysfunction induced by doxorubicin treatment. These findings provide overwhelming evidence that systemic inhibition of P2Y_6_R exacerbates pressure overload-induced heart failure in mice, although P2Y_6_R in cardiomyocytes contributes to the progression of cardiac fibrosis.

## Introduction

Cardiac remodeling is characterized by structural and morphological changes of the heart, including hypertrophy and fibrosis, and is a major clinical outcome of heart failure after cardiac injury^1,2^. Structural remodeling is thought to be a plasticity process of the heart to overcome hemodynamic overload, but cardiac resistance (i.e., robustness) to mechanical stress may be reduced by additional environmental factors, such as physical and chemical stresses^3^.

Purinergic receptors are activated by extracellular nucleotides and play important roles in cardiovascular physiology and pathophysiology^4^. Purinergic receptors are divided into two main groups, P1 and P2. P1 receptors are activated by adenosine, and mediate cardiodepressant and cardioprotective effects^4^. P2 receptors are subdivided into P2X and P2Y subfamilies, which consist of ligand-gated ion channels and G protein coupled receptors (GPCRs), respectively^4^. The P2Y family has eight subtypes (P2Y_1_, P2Y_2_, P2Y_4_, P2Y_6_, P2Y_11_, P2Y_12_, P2Y_13_ and P2Y_14_) that differ in their coupling G protein and ligand selectivity^5^. Purinergic signaling must be important for cardiovascular homeostasis because many purinergic receptors are expressed in human and mouse hearts^6,7^. The nucleotide, uridine triphosphate (UTP), induces a profibrotic response via P2Y_2_R^8^, while adenosine triphosphate (ATP) induces contraction^9^ and negatively regulates hypertrophic growth of cardiomyocytes^10,11^.

We have previously focused on the role of the uridine-responsive P2Y receptors, P2Y_2_R and P2Y_6_R, because they are upregulated in the mouse heart when exposed to pressure overload^7^. We have reported that treatment of rat cardiac fibroblasts with ATP downregulates angiotensin type 1 receptor (AT1R) through induction of inducible nitric oxide (NO) synthase^12^. P2Y_2_R also mediates ATP-induced suppression of cardiomyocyte hypertrophy^10^ and nutritional deficiency-induced cardiomyocyte atrophy^11^. P2Y_6_R, activated mainly by uracil diphosphate (UDP), changes the contractility of mouse cardiomyocytes^13^. Contractility of the aorta in response to UDP is different in P2Y_6_R-deficient mice compared with wild-type mice^14^. Therefore, P2Y_6_R may have an important role in cardiovascular contractility. In mouse aorta, P2Y_6_R levels are increased in an age-dependent manner and P2Y_6_R contributes to hypertensive vascular remodeling via its heterodimerization with AT1R^15^. In addition, P2Y_6_R has a deleterious role in atherosclerosis, being abundant in sclerotic lesions and promoting inflammation^16,17^. P2Y_6_R is also upregulated in pressure overloaded mouse hearts, and pharmacological inhibition of P2Y_6_R by MRS2578 attenuates pressure overload-induced cardiac fibrosis^7^. These findings indicate that P2Y_6_R in cardiovascular systems is a promising therapeutic target for cardiovascular dysfunction. However, it is not clear whether pressure overload-induced heart failure can be attenuated in P2Y_6_R-deficient mice. Indeed, deletion of P2Y_6_R in mice enhances isoproterenol-induced pathological cardiac hypertrophy^18^.

Several GPCRs, especially G_q_ protein-coupled receptors, are responsive to mechanical stress^19,20^. For example, AT1R, which is activated by angiotensin II, is directly activated by mechanical stretch without angiotensin II stimulation^21^. One of the major physiological roles of P2Y_6_R is to act as a mechano-activating GPCR in cardiomyocytes through ligand-dependent and -independent (AT1R-P2Y_6_R heterodimer-dependent) pathways^7,15^. However, whether these two mechano-activation mechanisms of P2Y_6_R have the same role is unknown. Therefore, we tested whether deletion of P2Y_6_R attenuates mechanical stress-induced cardiomyocyte hypertrophy in vitro. We demonstrate that knockdown of P2Y_6_R suppresses hypotonic stress-induced cell damage and hypertrophy in neonatal rat cardiomyocytes (NRCMs). However, P2Y_6_R hetero- and homo-deficient [P2Y_6_R^(+/−)^ and P2Y_6_R^(−/−)^] mice show vulnerability to pressure overload induced by transverse aortic constriction (TAC). In addition, cardiomyocyte-specific P2Y_6_R-expressing mice also show elevated pressure overload-induced cardiac fibrosis and contractile dysfunction. P2Y_6_R deficiency did not affect doxorubicin (DOX)-induced heart failure; therefore, systemic deletion of P2Y_6_R specifically augments cardiac vulnerability to mechanical stress.

## Results

### Knockdown of P2Y_6_R suppresses cell damage and hypertrophy induced by hypotonic stress in vitro

**Figure 1:**
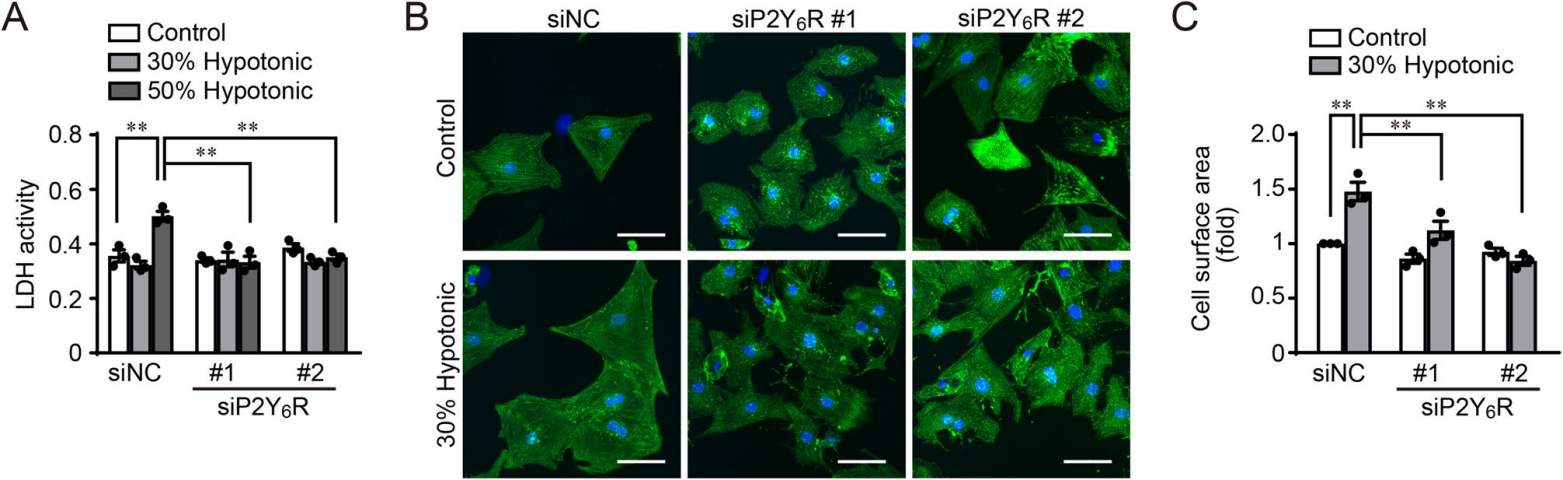
Knockdown of P2Y_6_R in NRCMs suppresses hypotonic stress-induced cell damage and hypertrophy. (**A**) Activity of lactate dehydrogenase (LDH) released from damaged cells. (*n* = 3 independent experiments). (**B**) NRCMs transfected with negative control (siNC) or P2Y_6_R (siP2Y_6_R #1 and #2) siRNA were immunostained with an anti-actinin antibody. (*n* = 3 independent experiments). Scale bars, 50 µm. (**C**) Anti-actinin-immunostained NRCM surface area. (*n* = 3 independent experiments). Data are shown as means ± SEM. **P < 0.01, one-way ANOVA.

We previously reported that selective antagonist inhibition of P2Y_6_R suppressed cardiac remodeling and dysfunction after pressure overload^7^. However, effects of P2Y_6_R deficiency on pressure overload-induced cardiac remodeling have not been investigated. We therefore knocked down P2Y_6_R in NRCMs using two siRNAs (siP2Y_6_R #1 and #2), and examined cell damage and size after hypotonic stimulation, which is a model of in vitro pressure overload. Cytotoxicity was analyzed by measuring the activity of lactate dehydrogenase released by damaged cells, and the size of α-actinin-positive NRCMs was also determined. Cell damage and hypertrophy induced by hypotonic stress were significantly suppressed in P2Y_6_R knockdown NRCMs (Fig. 1A–C). These data indicate that P2Y_6_R deficiency can be protective against pressure overload-induced cardiac remodeling and dysfunction. These results are consistent with those of a previous study, which showed that pharmacological inhibition of P2Y_6_R improves cardiac dysfunction after pressure overload^7^.

### Mice lacking P2Y_6_R died suddenly after pressure overload from exacerbated heart failure with enhanced cardiac hypertrophy

**Figure 2:**
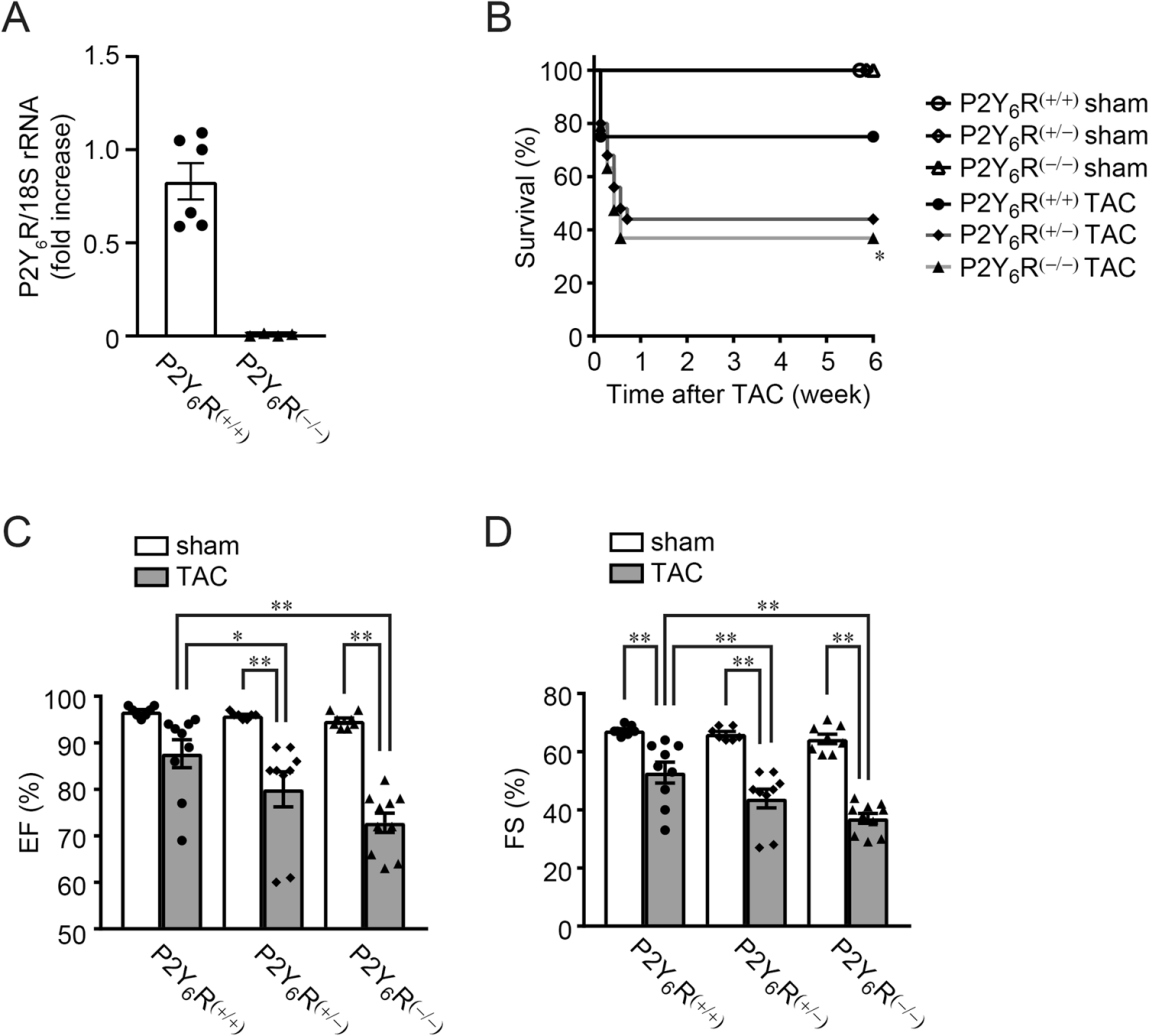
P2Y_6_R deficiency promotes pressure overload-induced heart failure. (**A**) The expression of P2Y_6_R in P2Y_6_R^(+/+)^ (*n* = 6) and P2Y_6_R^(−/−)^ (*n* = 4) mouse hearts was assessed by qPCR. Data are shown as means ± SEM. **P < 0.01, t-test. (**B**) Survival rate of P2Y_6_R^(+/+)^, P2Y_6_R^(+/−)^ and P2Y_6_R^(−/−)^ mice after TAC. (*n* = 10 to 25 mice per treatment). *P < 0.05 compared to P2Y_6_R^(+/+)^ TAC, Log-rank test. (**C**,**D**) Contractile function in P2Y_6_R^(+/+)^, P2Y_6_R^(+/−)^ and P2Y_6_R^(−/−)^ mice, 5 weeks after TAC. Ejection fraction (C) and fractional shortening (D) (*n* = 7 to 10 mice per treatment). Data are shown as means ± SEM. *P < 0.05, **P < 0.01, one-way ANOVA.

**Figure 3:**
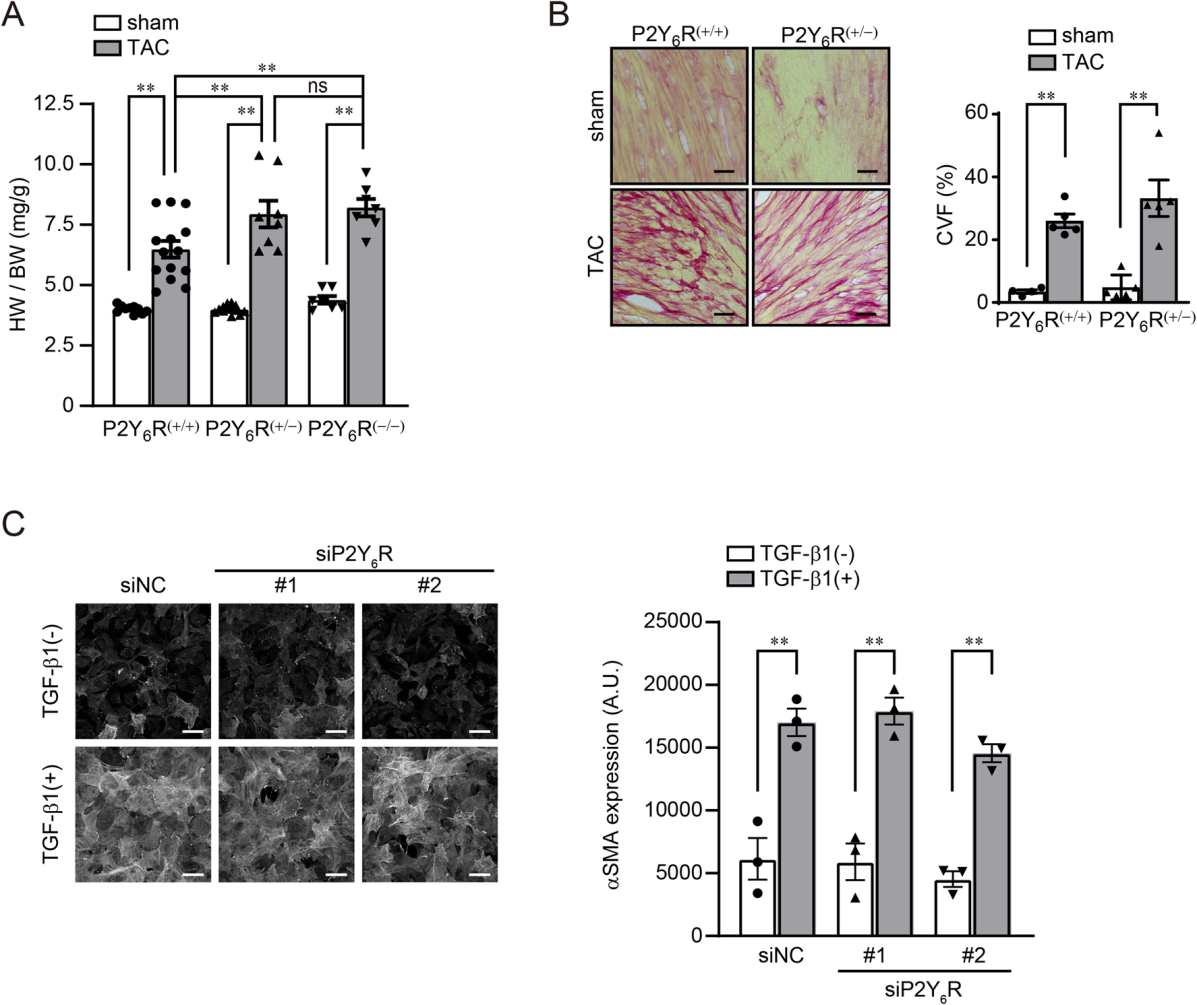
P2Y_6_R deficiency promotes hypertrophy of cardiomyocytes but not fibrosis after TAC. (**A**) Heart weight/body weight (HW/BW) ratio of P2Y_6_R^(+/+)^, P2Y_6_R^(+/−)^ and P2Y_6_R^(−/−)^ mice, 6 weeks after TAC. (*n* = 7 to 14 mice per treatment). (**B**) Cardiac collagen volume fraction of P2Y_6_R^(+/+)^ and P2Y_6_R^(−/−)^ mice 6 weeks after TAC. The frozen section was stained with Picrosirius red. (*n* = 4 to 5 mice per treatment). Scale bars, 50 µm. **C.** Neonatal rat cardiac fibroblasts were transfected with negative control (siNC) or P2Y_6_R (siP2Y_6_R #1 and #2) siRNA and immunostained with an anti-α-SMA antibody. (*n* = 3 independent experiments). Scale bars, 50 µm. Data are shown as means ± SEM. **P < 0.01, one-way ANOVA.

To confirm the effect of P2Y_6_R deletion in vivo, wild-type [P2Y_6_R^(+/+)^], P2Y_6_R hetero-deficient [P2Y_6_R^(+/−)^] and P2Y_6_R homo-deficient [P2Y_6_R^(−/−)^] mice were subjected to pressure overload by transverse aortic constriction (TAC) surgery. qPCR confirmed constitutive and systemic P2Y_6_R deletion (Fig. 2A). As previously reported, deletion of P2Y_6_R in mice does not alter fertility or growth and does not induce any organ abnormalities^14^. Indeed, we observed no phenotypic differences between P2Y_6_R^(+/+)^, P2Y_6_R^(+/−)^, and P2Y_6_R^(−/−)^ mice, including body weight, heart weight, and cardiac function, under homeostatic conditions. Surprisingly and in contrast to the expectation from the in vitro results, there was a rapid and high rate of death among P2Y_6_R^(+/−)^ and P2Y_6_R^(−/−)^ mice after TAC (Fig. 2B). The left ventricular contractile function was measured in surviving mice by echocardiography at 5 weeks after TAC (Table 1). Ejection fraction (EF) and fractional shortening (FS) was reduced in P2Y_6_R^(+/−)^ and P2Y_6_R^(−/−)^ TAC mice compared with that in P2Y_6_R^(+/+)^ TAC mice, whereas P2Y_6_R deficiency did not affect basal cardiac contractility (Fig. 2C,D). Cardiac hypertrophy and fibrosis were evaluated by heart weight/body weight ratio and picrosirius red staining, respectively. Cardiac hypertrophy (Fig. 3A) but not fibrosis (Fig. 3B) was enhanced in P2Y_6_R^(+/−)^ TAC and P2Y_6_R^(−/−)^ TAC mice compared with P2Y_6_R^(+/+)^ TAC mice. Consistent with echocardiography data (Fig. 2C,D), TAC-induced hypertrophy of P2Y_6_R^(+/−)^ mice was the same extent with that of P2Y_6_R^(−/−)^ mice (Fig. 3A), suggesting that heterozygous deletion of P2Y_6_R is critical and sufficient for TAC-induced cardiac dysfunction and remodeling. Fibroblast differentiation into myofibroblasts stimulates excessive ECM deposition, leading to cardiac fibrosis^22^. We therefore investigated whether P2Y_6_R deficiency affected TGF-β1-induced differentiation into myofibroblasts. TGF-β1 treatment of cardiac fibroblasts increased the expression of α-smooth muscle actin (α-SMA), a marker of differentiated myofibroblasts, and P2Y_6_R knockdown did not affect the expression of α-SMA after TGF-β1 treatment (Fig. 3C). Consistent with the in vivo result (Fig. 3B), P2Y_6_R deficiency did not affect the differentiation of fibroblasts into myofibroblasts. These data indicate that P2Y_6_R knockdown in vivo confers vulnerability to pressure overload, which is opposite to the in vitro results and to the findings of a previous inhibitor study^7^.

**Table 1:** Cardiac parameters measured by echocardiography 5 weeks after TAC in P2Y_6_R^(+/+)^, P2Y_6_R^(+/−)^ and P2Y_6_R^(−/−)^ mice.

	P2Y_6_R^(+/+)^ sham (n = 7)	P2Y_6_R^(+/−)^ sham (n = 7)	P2Y_6_R^(−/−)^ sham (n = 8)	P2Y_6_R^(+/+)^ TAC (n = 9)	P2Y_6_R^(+/−)^ TAC (n = 9)	P2Y_6_R^(−/−)^ TAC (n = 10)
IVSTd (mm)	0.871 ± 0.06	0.900 ± 0.05	0.763 ± 0.05	1.311 ± 0.10**^,††,‡‡^	1.344 ± 0.07**^,††,‡‡^	1.270 ± 0.06**^,††,‡‡^
LVIDd (mm)	2.986 ± 0.07	3.300 ± 0.07	3.213 ± 0.12	3.200 ± 0.12	3.489 ± 0.18*	3.170 ± 0.08
LVPWd (mm)	0.829 ± 0.06	0.771 ± 0.13	0.738 ± 0.04	1.267 ± 0.08**^,††,‡‡^	1.378 ± 0.07**^,††,‡‡^	1.322 ± 0.07**^,††,‡‡^
LVIDs (mm)	1.000 ± 0.05	1.114 ± 0.05	1.150 ± 0.1	1.522 ± 0.18*	2.000 ± 0.24**^,††,‡‡,^^##^	2.000 ± 0.08**^,^^††^^,^^‡‡^^,^^#^
EF	0.967 ± 0.00	0.959 ± 0.00	0.948 ± 0.01	0.877 ± 0.04	0.800 ± 0.05**^,^^††^^,^^‡‡^^,^^#^	0.728 ± 0.03**^,††,‡‡,^^##^^,§^
FS	0.673 ± 0.01	0.661 ± 0.01	0.644 ± 0.02	0.528 ± 0.04**^,††,‡‡^	0.439 ± 0.04**^,††,‡‡,^^##^	0.371 ± 0.02**^,††,‡‡,^^##^^,§^

IVSTd, interventricular septum thickness, diastolic; LVIDd, left ventricular internal dimension, diastolic; LVPWd, left ventricular posterior wall, diastolic; LVIDs, left ventricular internal dimension, systolic; EF, ejection fraction; FS, fractional shortening. Data are shown as means ± SEM.

*P < 0.05, **P < 0.01 versus P2Y_6_R^(+/+)^ sham, ^††^*P* < 0.01 versus P2Y_6_R^(+/−)^ sham, ^‡‡^*P* < 0.01 versus P2Y_6_R^(−/−)^ sham, ^#^*P* < 0.05, ^##^*P* < 0.01 versus P2Y_6_R^(+/+)^ TAC, ^§^*P* < 0.05 versus P2Y_6_R^(+/−)^ TAC, one-way ANOVA.

### Cardiomyocyte-specific overexpression of P2Y_6_R in mice also promoted cardiac dysfunction after pressure overload

**Figure 4:**
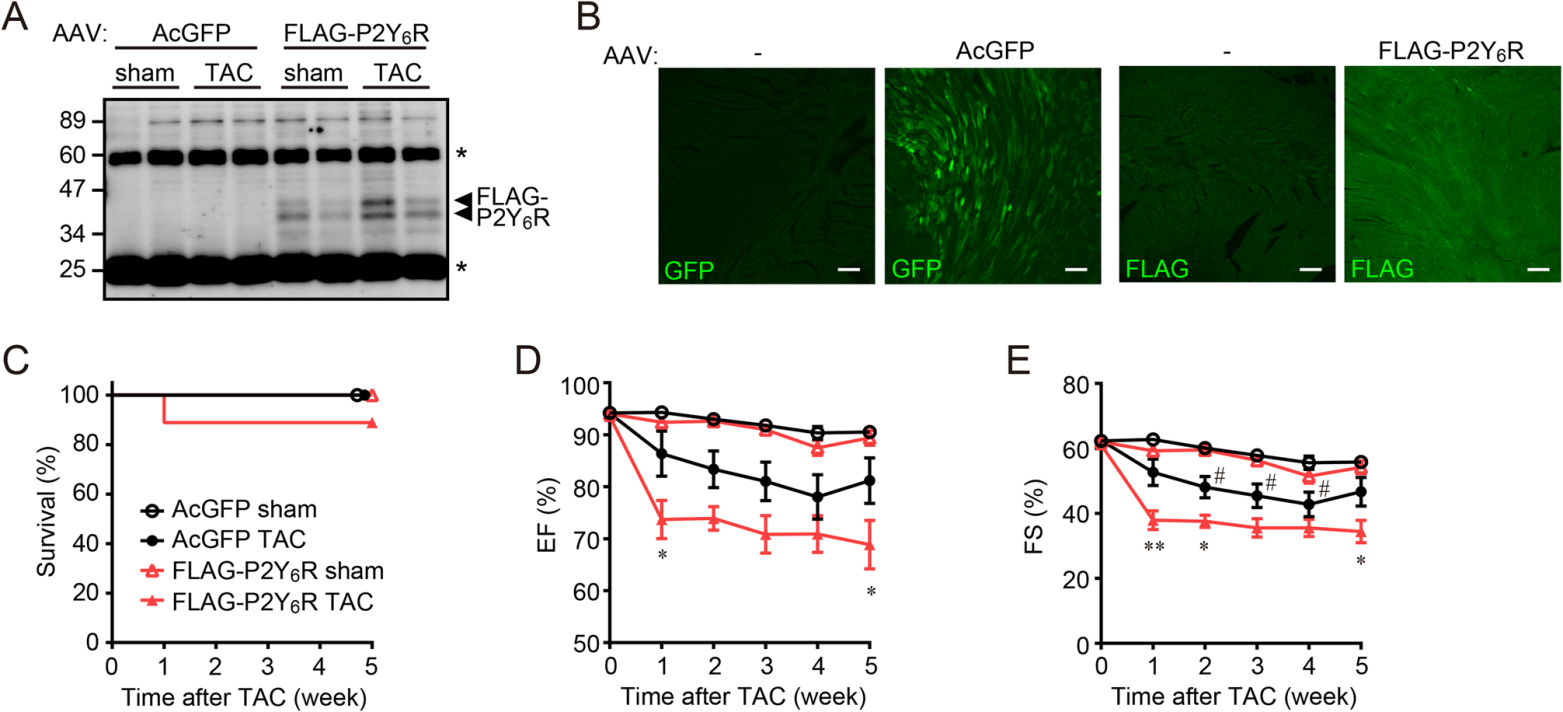
Cardiomyocyte-specific overexpression of P2Y_6_R promotes pressure overload-induced heart failure. (**A**,**B**) Overexpression of FLAG-P2Y_6_R proteins in cardiac tissue 13 weeks after adeno-associated viral (AAV) vector infection. Expression of FLAG-P2Y_6_R determined by western blotting of anti-FLAG immunoprecipitate from whole heart (A) and immunostaining of cardiac sections with anti-GFP or FLAG antibodies (B). (*n* = 3 mice per treatment). Scale bar, 100 µm. Asterisks show heavy and light chains of IgG. (**C**) Survival rate of control and P2Y_6_R-overexpression mice after transverse aortic constriction (TAC). (*n* = 5 to 9 mice per treatment). (**D**,**E**) Contractile function of control and P2Y_6_R-overexpression mice after TAC. Ejection fraction (**D**) and fractional shortening (**E**) (*n* = 5 to 9 mice per treatment). Data are shown as means ± SEM. ^#^*P* < 0.05 versus AcGFP sham, *P < 0.05, **P < 0.01 versus AcGFP TAC, two-way ANOVA.

**Figure 5:**
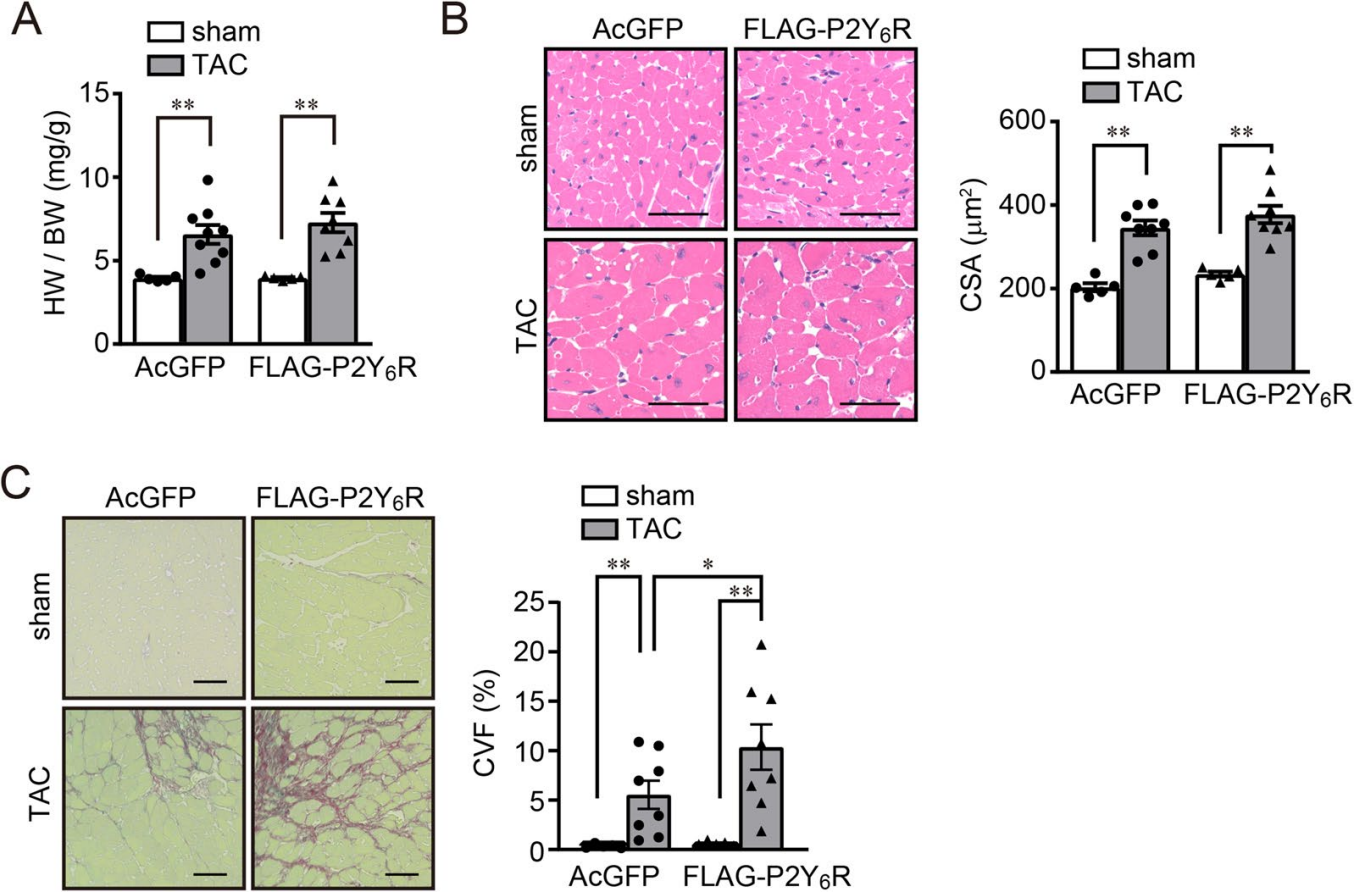
Cardiomyocyte-specific overexpression of P2Y_6_R promotes fibrosis but not hypertrophy of cardiomyocytes after transverse aortic constriction (TAC). (**A**) Heart weight/body weight (HW/BW) ratio of control and P2Y_6_R-overexpression mice after TAC. (*n* = 5 to 9 mice per treatment). (**B**) Cardiomyocyte cross sectional area of control and P2Y_6_R-overexpression mice 5 weeks after TAC was measured using hematoxylin–eosin staining. (*n* = 5 to 8 mice per treatment). Scale bar, 50 µm. (**C**) Cardiac collagen volume fraction of control and P2Y_6_R-overexpression mice 5 weeks after TAC. The paraffin section was stained with Picrosirius red. (*n* = 5 to 8 mice per treatment). Scale bar, 50 µm. Data are shown as means ± SEM. *P < 0.05, **P < 0.01, one-way ANOVA.

From previously published findings and the data shown in Figs. 2 and 3, we hypothesized that an adequate level of P2Y_6_R expression is important for an appropriate response against pressure overload. Therefore, to assess the effects of P2Y_6_R overexpression in pressure overloaded-heart, we generated mice with cardiomyocyte-specific P2Y_6_R overexpression using an adeno-associated viral (AAV) vector. AcGFP and FLAG-P2Y_6_R were successfully expressed in cardiac tissue by the AAV vector (Fig. 4A,B). The survival rate of mice with cardiomyocyte-specific overexpression of FLAG-P2Y_6_R (FLAG-P2Y_6_R mice) after TAC was not significantly different compared to that of AcGFP mice (Fig. 4C). This indicates that cardiomyocyte-specific overexpression of P2Y_6_R does not induce sudden death, which is different from P2Y_6_R knockdown (Fig. 2B). Cardiomyocyte-specific overexpression of P2Y_6_R did not affect basal contractility in the heart, whereas EF and FS were significantly decreased in FLAG-P2Y_6_R mice compared with control (AcGFP) mice after TAC (Fig. 4D,E and Table 2). In contrast to P2Y_6_R^(−/−)^ mice, morphological analysis showed more cardiac fibrosis but not hypertrophy in FLAG-P2Y_6_R mice (Fig. 5A–C). These data indicate that a suitable level of P2Y_6_R in cardiomyocytes may be important for mechanical sensation.

**Table 2:** Cardiac parameters measured by echocardiography 5 weeks after TAC in AcGFP and FLAG-P2Y_6_R mice.

	AcGFP sham (n = 5)	FLAG-P2Y_6_R sham (n = 5)	AcGFP TAC (n = 9)	FLAG-P2Y_6_R TAC (n = 8)
IVSTd (mm)	1.038 ± 0.02	1.054 ± 0.04	1.366 ± 0.04**^,††^	1.382 ± 0.04**^,††^
LVIDd (mm)	3.021 ± 0.04	2.897 ± 0.04	2.914 ± 0.15	3.272 ± 0.17
LVPWd (mm)	1.074 ± 0.02	1.005 ± 0.04	1.390 ± 0.05**^,††^	1.308 ± 0.04**^,††^
LVIDs (mm)	1.338 ± 0.01	1.332 ± 0.07	1.604 ± 0.22	2.184 ± 0.22*^,^^†^^,^^‡^
EF	0.905 ± 0.00	0.894 ± 0.01	0.812 ± 0.04	0.689 ± 0.05**^,††,‡^
FS	0.558 ± 0.01	0.542 ± 0.02	0.467 ± 0.04	0.344 ± 0.03**^,††,‡^

IVSTd, interventricular septum thickness, diastolic; LVIDd, left ventricular internal dimension, diastolic; LVPWd, left ventricular posterior wall, diastolic; LVIDs, left ventricular internal dimension, systolic; EF, ejection fraction; FS, fractional shortening. Data are shown as means ± SEM.

*P < 0.05, **P < 0.01 versus AcGFP sham, ^†^*P* < 0.05, ^††^*P* < 0.01 versus FLAG-P2Y_6_R sham, ^‡^*P* < 0.05 versus AcGFP TAC, one-way ANOVA.

### Non-mechano-induced heart failure was not affected by P2Y_6_R deficiency

**Figure 6:**
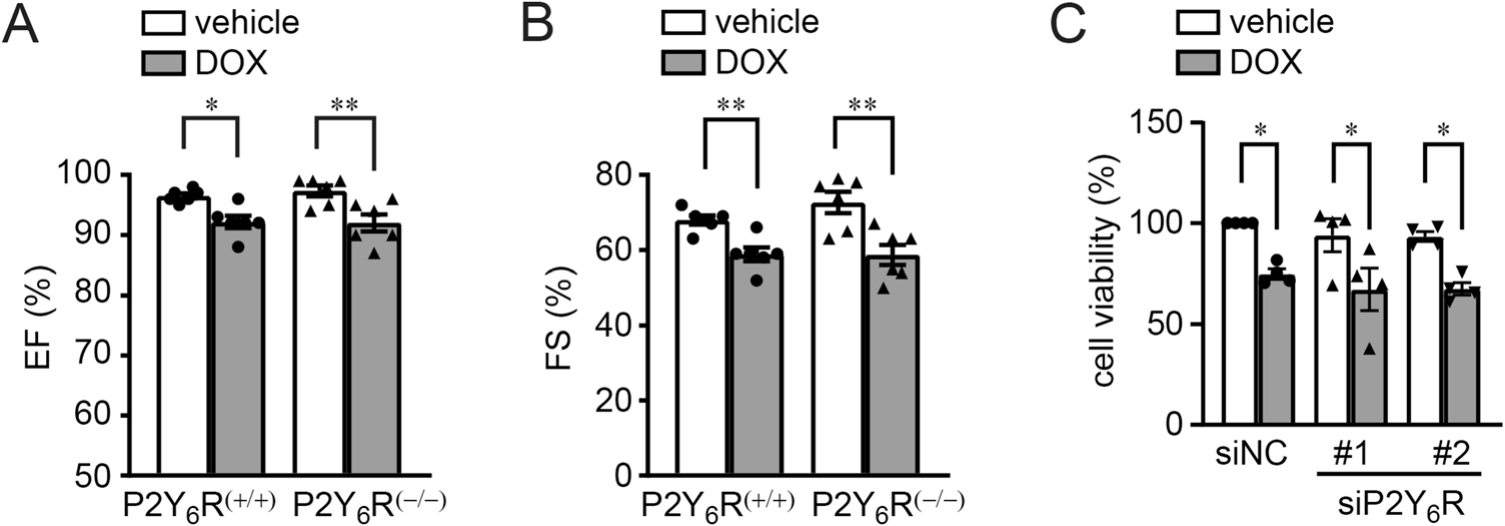
P2Y_6_R deficiency fails to exacerbate doxorubicin-induced heart failure. (**A**,**B**) Contractile function of P2Y_6_R^(+/+)^ and P2Y_6_R^(−/−)^ mice, 3 weeks after DOX injection. Ejection fraction (**A**) and fractional shortening (**B**) were measured by echocardiography. (*n* = 6 mice per treatment). (**C**) Cell viability of NRCMs was assessed by MTT assays. NRCMs were transfected with negative control (siNC) or P2Y_6_R (siP2Y_6_R #1 and #2) siRNA and treated with vehicle or DOX (*n* = 4 independent experiments). Data are shown as means ± SEM. *P < 0.05, **P < 0.01, one-way ANOVA.

Finally, we checked whether P2Y_6_R expression affects non-mechano-induced heart failure using a DOX-induced heart failure model^23^. DOX was administered to P2Y_6_R^(+/+)^ and P2Y_6_R^(−/−)^ mice and left ventricular contractile function was assessed by echocardiography. Left ventricular contractile function was similar among P2Y_6_R^(+/+)^ and P2Y_6_R^(−/−)^ mice after DOX infusion (Fig. 6A,B). Cardiomyocyte death is one of the major causes of DOX-induced cardiotoxicity, leading cardiac dysfunction^24^. P2Y_6_R knockdown did not affect DOX-induced death of NRCMs (Fig. 6C), indicating that P2Y_6_R deficiency does not affect cardiac remodeling induced by DOX. These data support P2Y_6_R expression affecting only mechano-induced-cardiac remodeling.

## Discussion

We previously reported that activation of P2Y_6_R promotes cardiac remodeling in a pressure overload model^7^. Consistent with this, cardiomyocyte-specific overexpression of P2Y_6_R increased pressure overload-induced heart failure with severe fibrosis (Figs. 4, 5). Additionally, deletion of P2Y_6_R in vitro significantly suppressed cell damage and hypertrophy after hypotonic stimulation (Fig. 1). From these results, we hypothesize that deletion of P2Y_6_R in mice is protective against pressure overload-induced cardiac remodeling and heart failure. Surprisingly, there was a high death rate among P2Y_6_R-deficient mice within 5 days after TAC surgery (Fig. 2B). In addition, cardiac contractile function was reduced and cardiac hypertrophy was increased after TAC. Cardiac fibrosis did not change compared with that in wild-type mice (Figs. 2, 3). The results of P2Y_6_R knockout mice appear to conflict with those of P2Y_6_R overexpression mice. To explain this discrepancy, we need to take the difference between these two models into consideration. In the overexpression model, P2Y_6_R expression was driven by the cardiomyocyte-specific cTnT promoter, and P2Y_6_R signaling was directly altered in cardiomyocytes only. In P2Y_6_R^(−/−)^ mice, P2Y_6_R is deleted in all cell types. The heart contains many cell types in addition to cardiomyocytes, including smooth muscle cells, endothelial cells, fibroblasts, connective tissue cells, mast cells, and immune system-related cells, and these cells cooperatively work together to maintain cardiac homeostasis^25^. Therefore, in vivo cardiac vulnerability caused by P2Y_6_R knockout may be caused by non-cardiomyocytes. Indeed, non-cardiomyocytes may contribute to hypertrophy and fibrosis^26^. For instance, inflammatory cells, such as macrophages and monocytes, infiltrate the heart in response to pressure overload and affect the hypertrophic response and pathogenesis of heart failure^27^. Deletion of activated cardiac fibroblasts suppresses fibrotic responses^28–30^. Moreover, purinergic signaling takes part in the inflammatory response^31^. Neutrophils, a type of inflammatory cell, release nucleotides during membrane deformation, which is important for neutrophil function^32^. Also in rat cardiac fibroblasts and neonatal rat cardiac myofibroblasts, many types of purinergic receptors are expressed^33^. Interestingly, the level of P2Y_6_R expression is selectively upregulated in endothelial cells after tumor necrosis factor α-stimulation^34^. Additionally, P2Y_6_R has critical roles in activating immune cells, such as macrophages and microglia in various tissues^16,35–37^. Based on these findings, our results concerning cardiac vulnerability in P2Y_6_R knockout and cardiomyocyte-specific P2Y_6_R overexpression mice after pressure-overload strongly indicate that P2Y_6_R signaling plays a pivotal role in a variety of cell types, including cardiomyocytes, fibroblasts and macrophages, and that this makes the relationship between P2Y_6_R and cardiovascular disease complex. Further study using cell type-specific P2Y_6_R knockout mice will be needed to elucidate the detailed mechanism.

The second possibility to explain this discrepancy is variety of P2Y_6_R activation modes. It has been recently reported about various modes of GPCR activation including biased activation and dimerization^38^. When GPCRs are activated, they can activate not only G protein-dependent but also β-arrestin-dependent signaling pathway. Moreover, in several situations, GPCR can selectively activate either G protein or β-arrestin signaling pathway, which is called biased activation. Interestingly, G protein and β-arrestin activate different downstream signaling molecules, leading different cardiac responses^39^. β1-adrenergic receptor-mediated G protein signaling pathway promotes harmful cardiac remodeling, whereas β-arrestin signaling pathway shows cardioprotective effect^40^. We previously identified that P2Y_6_R acts as G protein-biased modulator of angiotensin II type 1 receptor (AT1R)^15^. AT1R-P2Y_6_R heterodimerization converts AT1R signaling from β-arrestin to G protein pathway, leading vascular remodeling. P2Y_6_R is activated by extracellular UDP. We have previously reported that UDP-activated P2Y_6_R induces cardiac fibrosis through G_12/13_ pathway^7^. Interestingly, a non-nucleotide ligand for P2Y_6_R has recently been identified^41^. Moreover, several G_q_-coupled GPCRs act as mechanosensors and are directly activated by mechanical stretch^19^. Therefore, these multiple modes of P2Y_6_R activation including biased activation and non-canonical ligands may result in complex phenotypes on cardiac remodeling.

Deletion of P2Y_6_R has positive effects in atherosclerosis models^16,17^. However, as shown here, loss of P2Y_6_R increased pressure overload-induced heart failure. Moreover, isoproterenol-induced cardiac pathological hypertrophy is also augmented in P2Y_6_R-deficient mice^18^. This phenotypic difference between vascular and cardiac remodeling may depend on cell types. In an atherosclerosis model, agonist-induced P2Y_6_R activation causes leukocyte activation, such as rolling and adhesion^16^. In addition, P2Y_6_R signaling induces the release of inflammatory cytokines from macrophages^17^. These results indicate that P2Y_6_R-mediated pro-inflammatory responses in immune cells such as leukocytes and macrophages are primary contributors to atherosclerosis formation. However, in isoproterenol-induced hypertrophy, P2Y_6_R signaling suppresses isoproterenol-induced cardiomyocyte hypertrophy^18^ and this signaling may occur in cardiomyocytes.

DOX-induced heart failure is mainly caused by oxidative stress and not by mechanical stress^23^. The mechanism of cardiac remodeling induced by DOX is different from that induced by TAC. TAC induces myocardial hypertrophy and fibrosis, whereas DOX induces cardiac atrophy^23^ and cell death^24^. In this study, P2Y_6_R deficiency did not affect cardiac contractility after doxorubicin injection in vivo (Fig. 6A,B) or cell death of DOX-treated cardiomyocytes in vitro (Fig. 6C), indicating that P2Y_6_R does not participate in oxidative stress-induced cardiac injury, but specifically contributes to mechanical stress-induced heart failure.

In addition to TAC and DOX injection, administration of isoproterenol or angiotensin II is used in rodent heart failure models. In isoproterenol-induced heart failure models, deletion of P2Y_6_R exacerbates cardiac hypertrophy but not fibrosis^18^. These data are similar to our results in the pressure overload-induced heart failure model. There is no evidence that shows whether P2Y_6_R affects angiotensin II-induced heart failure. However, P2Y_6_R can interact with AT1R, and deletion of P2Y_6_R suppresses angiotensin II-induced hypertension and vascular remodeling^15^. P2Y_6_R may also participate in angiotensin II-induced cardiac remodeling.

The mortality of wild-type TAC mice presented in Figs. 2 and 4 was different. This difference may be caused by AAV infection because AAV infection induces an immune response^42^, which can alter cardiovascular homeostasis. In addition, the anesthetic inhalation protocol was different for mice with/without AAV infection because AAV infection altered the response to the anesthetic.

In conclusion, although P2Y_6_R in cardiomyocytes definitely plays a key role in mechanical stress-induced cardiac remodeling, our results provide solid evidence that systemic inhibition of P2Y_6_R fails to prevent heart failure after pressure overload in mice. Strategies targeting P2Y_6_R for the treatment of heart failure will need careful consideration.

A limitation of this study is that echocardiography was used to assess cardiac function. However, the detected contractile function in healthy mice was high (EF = 90–95%) compared with a report using MRI (EF = approximately 60%)^43^. This difference is caused by the resolution of our echocardiography device being lower than that of MRI and of echocardiography devices that are specialized for small animal experiments. In addition, M-mode echocardiography is two-dimensional and EF is a calculated parameter. In contrast, MRI can yield three-dimensional information and measure EF directly. Unexpectedly, many P2Y_6_R knockout mice died after pressure-overload, and we could only obtain a minimal number of samples. According to the principle of the 3Rs, we used the minimum number of samples for analysis. Therefore, several lines of evidence were obtained from in vitro experiments only (such as Fig. 3C).

## Methods

### Animals

All experiments using rodents were reviewed and approved by the ethics committees at the National Institutes of Natural Sciences and carried out in accordance with their guidelines (Inter-University Research Institute Corporation National Institutes of National Sciences Animal Experiment Regulations). C57BL/6J mice and Sprague–Dawley (SD) rats were purchased from SLC. Animals were maintained under a 12-h/12-h light/dark cycle. P2Y_6_R^(−/−)^ mice (a kind gift from Professor Bernard Robaye, (Universite´ Libre de Bruxelles)) were backcrossed onto a C57BL/6J background to obtain P2Y_6_R^(+/−)^ mice as described previously^14^. Eight-week-old male mice were used for experiments.

### Isolation of neonatal rat cardiomyocytes and fibroblasts and transfection

Neonatal rat cardiomyocytes (NRCMs) and fibroblasts (NRCFs) were isolated from SD rat pups (1 to 2 days old) as previously described^11,44^. For knockdown, NRCMs and NRCFs were transfected with siRNA (20 nM) using Lipofectamine RNAiMAX Transfection Reagent (Invitrogen). siRNAs for rat P2Y_6_R (#1: P2ry6RSS300847, #2: P2ry6RSS300848) were purchased from Invitrogen.

### Measurement of cell damage

Activity of lactate dehydrogenase (LDH) released from damaged cells was measured using the Cytotoxicity LDH assay kit WST (Dojindo). Absorbance at 490 nm was measured using a SpectraMax i3 plate reader (Molecular Devices). To induce hypotonic stimulation, NRCMs were incubated in control, 30%, or 50% hypotonic solution (70% DMEM and 30% distilled water, and 50% DMEM and 50% distilled water, respectively).

### Measurement of cell surface area

NRCMs were seeded on 12φ glass base dishes (Iwaki) coated with Matrigel (Corning). After control or 30% hypotonic stimulation, NRCMs were fixed in 4% paraformaldehyde (FUJIFILM Wako) for 10 min, then permeabilized and blocked in 0.05% Triton X-100 with 1% bovine serum albumin (Nakalai Tesque) for 30 min. Anti-sarcomeric α-actinin (EA-53) (Abcam) was used as a primary antibody overnight at 4℃. Alexa-Fluor488-conjugated antibody (Life Technologies) was used as a secondary antibody. Stained NRCMs were mounted with ProLong Diamond Antifade Mountant containing DAPI (Invitrogen). Imaging was performed on a BZ-X700 microscope (KEYENCE). Cell surface area was measured using ImageJ. More than one hundred cells from five pictures were quantified for each specimen.

### Cardiac fibroblast differentiation

NRCFs were seeded on 12φ glasses. After treatment of 10 ng/mL TGF-β1, NRCFs were fixed in 4% paraformaldehyde for 10 min, then permeabilized and blocked in 0.05% Triton X-100 with 1% bovine serum albumin for 30 min. To check differentiation of NRCFs, anti-α-SMA antibody (Sigma) was used as a primary antibody overnight at 4˚C. Alexa-Fluor488-conjugated antibody was used as a secondary antibody. Stained NRCFs were mounted with ProLong Diamond Antifade Mountant containing DAPI. Imaging was performed on a Nikon A1Rsi microscope (Nikon). Expression level of α-SMA was measured using ImageJ. Five pictures were quantified for each specimen.

### Transverse aortic constriction (TAC) surgery

To induce cardiac pressure overload, TAC surgery was performed. The procedure was optimized, based on a previous study^45^. Briefly, male mice were anesthetized with isoflurane (abbvie), then intubated and ventilated. The chest cavity was opened at the intercostal area. Then, the transverse aorta was constricted with a 7–0 nylon suture between the brachiocephalic artery and the left carotid artery to the width of a 27-gauge needle. After closing the chest cavity, buprenorphine was administrated intraperitoneally as an analgesic.

### Measurement of cardiac functions

Echocardiography was performed as described previously^45^. Mice were anesthetized with isoflurane and cardiac function was measured by Nemio-XG echocardiography (Toshiba) with a 14 MHz imaging transducer.

### Morphological analysis

Mouse hearts were harvested, washed in PBS, and fixed in 10% neutral-buffered formalin (Nacalai Tesque). Fixed cardiac tissues were embedded in paraffin and sectioned. For frozen sections, hearts were embedded in O.C.T. compound (Sakura Finetek) and snap-frozen with liquid nitrogen. For measurement of cross sectional cardiomyocyte areas, hematoxylin/eosin staining was performed. For collagen volume fraction, Picrosirius red staining was conducted. Quantification of cardiomyocyte cross sectional area and collagen volume fraction were performed using ImageJ software.

### Cardiomyocyte-specific overexpression of P2Y_6_R in mice

All experiments using recombinant DNA were reviewed and approved by the safety committees for recombinant DNA of the National Institute for Physiological Sciences and were performed in accordance with their guidelines (National Institute for Physiological Sciences Recombinant DNA Experiment Rules). Adeno-associated viral (AAV) vectors encoding cardiomyocyte-specific expressed AcGFP and FLAG-tagged P2Y_6_R (FLAG-P2Y_6_R) under control of cardiac troponin T were generated as described previously^46^. AAV vectors were injected intraperitoneally into 7-day-old C57BL/6J male mice. Expression was examined by western blotting using an anti-FLAG M2 horseradish peroxidase-conjugated antibody (Sigma) following immunoprecipitation using anti-FLAG M2 beads (FUJIFILM Wako) and by immunofluorescence using an anti-FLAG antibody (Sigma). For western blotting, cardiac tissue was harvested and lysed in cell lysis buffer [20 mM HEPES (pH 7.4), 100 mM NaCl, 3 mM MgCl_2_, and 1% Triton X-100] with protease inhibitor cocktail (Nacalai Tesque) and Phosstop (Roche) to extract proteins. SDS-PAGE was performed in 12% polyacrylamide gels and proteins were then transferred to PVDF membranes. Proteins on membranes were reacted with antibodies and detected using the ImageQuant LAS4000 system (GE).

### Administration of doxorubicin (DOX)

15 mg/kg DOX (Sand) in saline was administrated intravenously to P2Y_6_R^(+/+)^ and P2Y_6_R^(−/−)^ mice on the first day of the experiment. Cardiac function after DOX administration was monitored by echocardiography.

### Statistical analysis

The results are presented as means ± SEM from at least three independent experiments. Statistical comparisons were performed by two-tailed Student’s t-test (for two groups) or one-way analysis of variance followed by a Newman-Keuls comparison procedure (for three and more groups). Significance was accepted for values of *P* < 0.05.
